# Synergistic Enhancement of Electrochemical-Oxidative Chlorine-Free Bromine Extraction from Oil and Gas Field Water by Zero-Gap Electrolyzer and Carbon Cloth Electrode: A Study on Efficient, Selective Extraction and Resistance to Other Ions

**DOI:** 10.3390/ma19050850

**Published:** 2026-02-25

**Authors:** Shiyong Zhou, Rong Ji, Yuan Li

**Affiliations:** 1 Hubei Key Laboratory of Mineral Resources Processing and Environment, Key Laboratory of Green Utilization of Critical Non-Metallic Mineral Resources, Ministry of Education, Wuhan University of Technology, 122 Luoshi Road, Wuhan 430070, China; 18348499089@163.com; 2 School of Metallurgy and Environment, Central South University, Changsha 410083, China; jirong@sinochem.com

**Keywords:** electrooxidative bromine extraction, selective oxidation, carbon cloth electrode, zero-gap electrolyzer, oil and gas field water

## Abstract

**Highlights:**

**What are the main findings?**

- Zero-gap electrolyzer with carbon cloth achieves >90% Br^−^ oxidation in 12 min.
- Selective Br^−^ oxidation realized at 1.0–1.52 V, avoiding Cl_2_ generation.
- Low Cl^−^ promotes Br^−^ oxidation, high Cl^−^ inhibits via adsorption competition.
- CO_3_^2−^ weakly promotes, Ca^2+^/Mg^2+^ negligible; organics (>80 mg/L) severely hinder.

**What are the implications of the main findings?**

- Enables safe, chlorine-free bromine extraction from oilfield brine.
- Zero-gap design enhances kinetics and efficiency for industrial scalability.
- Guides optimization of parameters and pretreatment strategies for real brines.
- Provides framework for selective ion recovery from complex saline matrices.

**Abstract:**

Bromine, as a strategic fundamental chemical raw material, is crucial for modern industry, but the traditional chlorine displacement method poses safety risks in oilfield brine development and faces challenges like resource depletion and inefficient utilization. Addressing the need for high-concentration bromine brine development in underground oilfields, this study developed an electrochemical oxidation-based chlorine-free bromine extraction technology. Leveraging the standard redox potential difference between Br^−^ and Cl^−^ (0.271 V), the effective potential window for selective Br^−^ oxidation was determined as 1.0–1.52 V (vs. SHE) via linear sweep voltammetry (LSV). Within this window, efficient and preferential oxidation of Br^−^ over Cl^−^ and OH^−^ was achieved. In simulated brine with high chloride and low bromide concentrations, a Br^−^ conversion rate of 92.3% was attained with no Cl_2_ generation. The self-designed zero-gap electrolyzer with carbon cloth as the anode reduced the reaction time by over 75% compared to a traditional H-type cell, oxidizing over 90% of Br^−^ within 12 min. Kinetic studies revealed that the reaction follows first-order kinetics, with current intensity positively correlated with Br^−^ concentration. Investigation of coexisting ions revealed that low concentrations of Cl^−^ promote the reaction, while high concentrations exert inhibitory effects. CO_3_^2−^ exhibits a weak promoting effect, and Ca^2+^/Mg^2+^ show negligible impact. Notably, organic matter (e.g., ethylene glycol) concentrations exceeding 80 mg/L substantially compromise bromine recovery efficiency. This technology provides a feasible solution for the safe and green development of high-concentration bromine resources and holds significant importance for the upgrading of the bromine chemical industry.

## 1. Introduction

As a strategic fundamental chemical raw material, bromine is widely used in critical fields such as pharmaceutical synthesis, pesticide preparation, flame retardant materials, and oil and gas extraction. The stable supply of bromine plays an indispensable role in the development of modern industry [1,2]. However, the self-sufficiency rate of bromine resources in most countries has long been insufficient, so there is an urgent need to develop new types of bromine resources and establish an efficient bromine extraction technology system.

Underground brine resources have long been the core raw material for bromine production. Nevertheless, long-term excessive exploitation and inefficient utilization have led to severe resource depletion. For instance, in the Shandong Peninsula region of China, the bromide ion (Br^−^) concentration in underground brine has declined from approximately 1500 ppm in the 1950s to the current 600 ppm, with some areas even falling below 400 ppm. This has significantly reduced the economic viability of bromine extraction [3,4,5,6]. In the exploration of new bromine resources, seawater contains a vast total amount of bromine, but its bromide ion concentration is only about 65 ppm. This extremely low concentration results in high energy consumption, poor economic efficiency, and limitations for large-scale development as well [7,8,9]. Recent research has revealed that bromide ion concentration in some underground oil and gas field water can be enriched up to 2 g/L, demonstrating substantial development potential [10,11]. The exploitation of such high-concentration bromine resources is expected to become a crucial breakthrough in alleviating the supply-demand imbalance of bromine.

Currently, the mainstream bromine extraction technology in the industry remains the chlorine displacement method. Its core principle relies on the strong oxidizing power of chlorine gas to oxidize bromide ions to Br_2_ [12,13,14]. However, chlorine gas readily reacts violently with the flammable components in oilfields and may even trigger explosions [15,16,17]. Therefore, using this method for bromine extraction in oilfields poses extremely high safety risks [18,19,20,21].

To address these issues, electrochemical oxidation technology has gradually emerged as a research hotspot due to its clean, efficient, and easily controllable characteristics [22,23,24]. The core theory of electrochemical chlorine-free bromine extraction lies in utilizing the standard redox potential difference between Br^−^ and Cl^−^ to achieve selective oxidation. The standard electrode potentials for the relevant reactions are as follows [25]:

Br_2_(aq) + 2e^−^ → 2Br^−^      E_0_ = 1.087 V (vs. SHE) (1)

Cl_2_(aq) + 2e^−^ → 2Cl^−^      E_0_ = 1.358 V (vs. SHE) (2)

The standard reduction potential of bromide ion (Br^−^) is 0.271 volts lower than that of chloride ion (Cl^−^) (versus the Standard Hydrogen Electrode, SHE). This implies that when the anode potential is controlled within the range of 1.087–1.358 V (vs. SHE), Br^−^ can theoretically be selectively oxidized while Cl^−^ remains stable. This potential window establishes the thermodynamic feasibility foundation for electrochemical chlorine-free bromine extraction technology. Izaak Cohen et al. proposed a method using a hybrid physical adsorption and capacitive deionization cell for bromine ion removal and recovery via electrooxidation and electroreduction, successfully achieving the removal and recovery of 3.5 mmol bromide ions using a working electrode containing 1 g of activated carbon cloth [26]. Zhang X et al. employed a three-electrode system and an electrolytic cell to achieve selective oxidation of bromide for bromine extraction from seawater [27,28]. However, the traditional H-type electrolytic cell features slow electron transfer, and noble metal electrodes exhibit weak adsorption capacity for bromide ions (Br^−^). These drawbacks make the efficiency of electro-oxidative bromine extraction under low voltage unable to meet the requirements of industrialization. Therefore, it is necessary to redesign a more efficient electrolytic cell and an electrode with stronger adsorption capacity for bromide ions, as well as explore the optimal operating voltage and the impact of other impurities in brine on the efficiency of electro-oxidative bromine extraction. Ultimately, this aims to achieve efficient and highly selective bromine extraction for industrial applications.

This study, in response to the unique endowments (high Cl^−^, low Br^−^) and safe development needs of oil and gas field water, focuses on the innovation of chlorine-free selective electrooxidative bromine extraction technology. By designing a novel zero-gap electrolyzer and optimizing reaction parameters, the selective electrooxidation mechanism of Br^−^ is systematically investigated. Additionally, the feasibility and performance of this technology in treating concentrated brine, as well as the influences of coexisting ions and organic substances, are verified. This study aims to establish a highly efficient, safe, and low-energy-consuming chlorine-free selective electrooxidative bromine extraction process, thereby laying a theoretical foundation for bromine extraction from oil and gas field water and supporting its scalability in industrial applications.

## 2. Materials and Methods

### 2.1. Electrode Materials

Two electrode systems were employed: a three-electrode system for linear sweep voltammetry (LSV) and chronoamperometric tests, and a two-electrode zero-gap electrolyzer for bromine extraction performance verification. In the three-electrode system, the working electrode (WE) for Br^−^ oxidation was carbon cloth (geometric area 2 cm × 1 cm, projected area 2 cm^2^); the counter electrode providing electron transfer was a platinum sheet with identical area; the reference electrode was a saturated KCl Ag/AgCl electrode for potential calibration. In the two-electrode system, the anode was the same carbon cloth as the WE, and the cathode was a platinum-plated titanium mesh with matching geometric and projected areas (2 cm × 1 cm, 2 cm^2^).

All electrodes underwent pretreatment to remove impurities and enhance activity. Carbon cloth was ultrasonically cleaned in absolute ethanol for 30 min, electrochemically activated in 0.5 mol/L H_2_SO_4_ at 1.5 V vs. SHE for 30 min, rinsed with deionized water to neutrality, and vacuum-dried at 60 °C for 2 h. Platinum-plated titanium mesh was soaked in 10% HCl for 1 h, rinsed with deionized water three times, and air-dried at 25 °C. Platinum sheet was polished with 0.3 μm alumina powder, ultrasonically cleaned in deionized water for 15 min, and dried with nitrogen.

### 2.2. Chemical Reagents

Sodium chloride (NaCl), sodium bromide (NaBr), sodium sulfate (Na_2_SO_4_), calcium chloride (CaCl_2_), magnesium chloride (MgCl_2_), hydrochloric acid (HCl), sulfuric acid (H_2_SO_4_), carbonic acid(H_2_CO_3_), and were procured from Sinopharm Chemical Reagent Co., Ltd. (Shanghai, China). All reagents can be used directly without further processing.

### 2.3. Data Processing

The concentration of Br^−^ in the electrolyte was determined using an ion chromatograph (IC, ICS2000, Dionex, Sunnyvale, CA, USA). The bromine yield was calculated according to the following equation [25]:

(3) $$\mathrm{Yield}=\frac{c_{{\mathrm{Br}}^{-},\mathrm{initial}}{-c}_{{\mathrm{Br}}^{-},\mathrm{end}}}{c_{{\mathrm{Br}}^{-},\mathrm{initial}}}$$

The electrooxidation process was primarily controlled by the electrochemical workstation. Current efficiency (η) and energy consumption (E) are key performance parameters, calculated using the following equations [25]:

(4) $$\eta \;=\;\frac{{\mathrm{FV}(c}_{{\mathrm{Br}}^{-},\mathrm{initial}}-c_{{\mathrm{Br}}^{-},\mathrm{end}})}{M\int_{0}^{t}\mathrm{Idt}}\times 100\%$$

(5) $$E=\frac{\int_{0}^{t}\mathrm{UIdt}}{\left(c_{{\mathrm{Br}}^{-},\mathrm{initial}}-c_{{\mathrm{Br}}^{-},\mathrm{end}}\right)V}\left(J/g\right)$$

where I is the cell current (A), t is the reaction time (s), F is the Faraday constant (96,485 C/mol), V is the volume of the electrolyte in the anode compartment (L), M is the molecular weight of the bromide salt (79.9 g/mol), U is the cell voltage (V), c_Br_^−^_,initial_ and c_Br_^−^_,end_ represent the initial and final molar concentrations of bromide ions (mol/L), respectively.

## 3. Results

### 3.1. Study on the Kinetic Characteristics of the Electrooxidation Chlorine-Free Bromine Extraction Process

To reveal the kinetic characteristics of the electrooxidative chlorine-free bromine extraction process, this study first constructed an electrochemical reaction system using an H-type electrolytic cell (Figure S1). The specific experimental design and procedures are as follows: the working electrode was a carbon cloth electrode (dimensions: 2 cm × 1 cm), the counter electrode was a platinum sheet electrode (dimensions: 2 cm × 1 cm), and the reference electrode was an Ag/AgCl electrode with a saturated KCl solution as the internal filling electrolyte. All measured potentials were calibrated to the standard hydrogen electrode (SHE) using the formula: E_SHE_ = E_Ag/AgCl_ + 0.197 V (25 °C). A H_2_SO_4_ solution at pH 3 was used as the solvent to prepare NaBr electrolytes with Br^−^ concentrations of 0.5 g/L, 4 g/L, and 8 g/L, respectively.

For the experiment of bromine extraction by electro-oxidation, 300 mL of the above electrolyte solutions with different Br^−^ concentrations was placed in a 500 mL beaker, and the tests were performed using a CHI 660C electrochemical workstation. To ensure the homogeneity of the electrolyte concentration, the solution was continuously stirred at room temperature using a magnetic stirrer at a rotation speed of 300 rpm, and the scan rate was set to 5 mV/s. In addition, chronoamperometric experiments were conducted using simulated brine as the reaction medium to analyze the dynamic relationship between the current intensity and the Br^−^ concentration.

**Figure 1:**
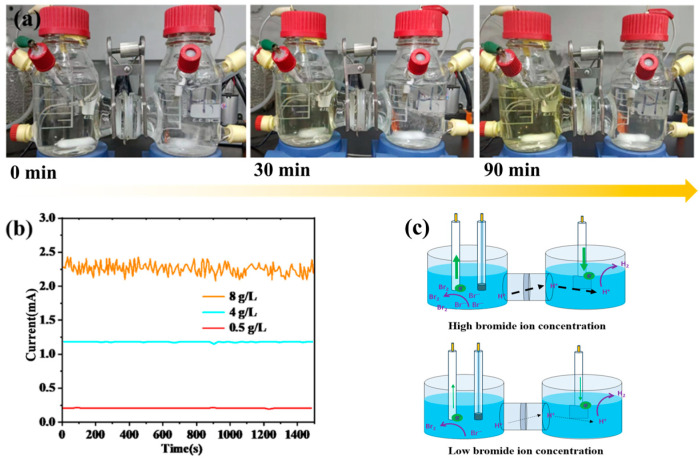
(**a**) Electrooxidative chlorine-free bromine extraction under a Br^−^ concentration of 8 g/L; (**b**) Current–time curves with varying Br^−^ concentrations; (**c**) Schematic diagram of the Br^−^ electrooxidation reaction in H-type electrolytic cell.

The experimental results are presented in Figure 1a,b. Under constant potential conditions, the reaction current intensity exhibited a significant positive correlation with the Br^−^ concentration. Within the 1500 s monitoring period, the average current was 2.336 mA for the 8 g/L Br^−^ solution, 1.247 mA for the 4 g/L Br^−^ solution, and only 0.248 mA for the 0.5 g/L Br^−^ solution. This phenomenon indicates that a higher Br^−^ concentration can provide more active reaction species (Figure 1c), thereby accelerating the electron transfer process at the electrode/electrolyte interface and ultimately leading to an enhancement in the reaction current intensity. Based on the fitting analysis of the above experimental data, the current control equation was derived as follows:

(6) $$y=2\times 10^{-5}\times x-10^{-4}\times x-6\times 10^{-5}$$

where *x* is Br^−^ concentration in g/L, y is current in A.

Based on the current control equation derived above, a quantitative correlation between real-time current monitoring and bromide ion (Br^−^) concentration can be established, thereby enabling the indirect calculation of Br^−^ concentration. This method provides a convenient and non-invasive technical approach for the dynamic tracking of Br^−^ concentration in the system. Furthermore, a detailed analysis of the kinetic behavior of this electrooxidative bromine extraction reaction revealed that its reaction kinetics conform to the characteristics of a pseudo-first-order reaction, with the reaction rate gradually decreasing as Br^−^ is continuously reacted [29,30]. This kinetic behavior not only provides a quantitative basis for the optimization of key parameters in the bromine extraction process, but also establishes a theoretical foundation for the real-time and precise monitoring of the reaction progress.

### 3.2. Zero-Gap Electrolyzer Performance and Parameter Optimization

Aiming at the bottlenecks in industrial application of the traditional H-type electrolytic cell during the electrooxidative bromine extraction process, this study verified its efficiency shortcomings through experiments: under a constant voltage of 1.35 V (vs. SHE), electrooxidation treatment was conducted on a simulated brine system with pH = 3 (containing 6 g/L Br^−^ and 120 g/L Cl^−^). The results showed that after 90 min of continuous reaction, the Br^−^ conversion rate was only slightly higher than 50%. Meanwhile, the current intensity remained at a low level of mA magnitude throughout the entire reaction cycle, directly leading to a significantly low reaction rate. This makes it difficult to meet the requirements for treatment efficiency in actual production and has become a key bottleneck restricting the large-scale application of this type of electrolytic cell in the electrooxidative bromine extraction process.

Based on the derivation and analysis of the theoretical formula for the electrolytic cell current, the current intensity (I) in the circuit is quantitatively related to the characteristics of the electrode material, the electrode cross-sectional area, and the proton exchange membrane cross-sectional area. The progress of the Br^−^ electrooxidation reaction is directly governed by this current behavior. The above relationship can be quantitatively described by the following electrolytic cell current formula [25]:

(7) $$I\;=\sqrt{\frac{K_{t}\times M\times S\times \tau }{\rho_{0}\times \left(1+\alpha \theta \right)}}$$

where I is the current flowing through the conductor, with the unit of ampere (A), K_t_ is the comprehensive heat dissipation coefficient, with the unit of watt per square meter per degree Celsius (W·m^−2^·°C^−1^), M and S are the cross-sectional perimeter and cross-sectional area of the electrode, respectively, where M has the unit of meter (m) and S has the unit of square meter (m^2^), τ is the temperature rise at the electrode surface (defined as the difference between the electrode surface temperature θ_0_(°C) and the ambient temperature θ_0_(°C), with the unit of degree Celsius (°C), ρ_0_ is the resistivity of the electrode material at 0 °C, with the unit of ohm meter (Ω·m), and α is the temperature coefficient of resistance of the electrode material, with the unit of per degree Celsius (°C^−1^).

To achieve the targeted optimization of electrooxidative bromine extraction performance, this study takes the aforementioned electrolytic cell current formula as the theoretical basis. Focusing on the correlation characteristics between current intensity (I), electrode cross-sectional perimeter (M), electrode cross-sectional area (S), and mass transfer efficiency in the formula, a systematic design was carried out from three aspects: electrode structure, electrolytic cell configuration, and material corrosion resistance.

**Figure 2:**
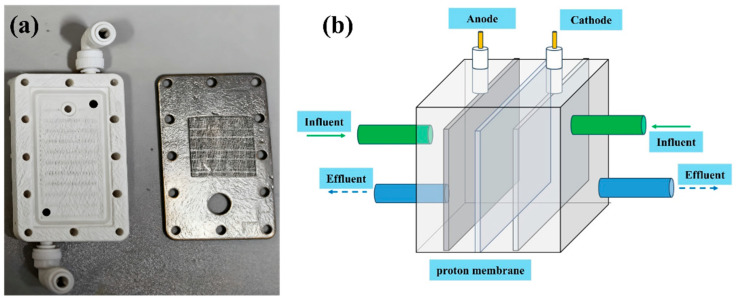
(**a**) The serpentine water channel structure and (**b**) Zero Gap electrolytic cell.

First, a large-area carbon cloth was selected as the anode material. By increasing the electrode’s cross-sectional perimeter (M) and effective cross-sectional area (S), the contact interface area between the electrode and electrolyte was expanded, providing a structural foundation for current enhancement. Second, the internal spatial layout of the electrolytic cell was optimized, and a Zero Gap configuration electrolytic cell was designed. This significantly shortened the proton transfer distance between the electrode and membrane, reducing mass transfer resistance. Meanwhile, a serpentine water channel structure was integrated into the cell to extend the residence time of incoming water in the electrolytic cell (Figure 2a), enhancing the probability of contact reactions between Br^−^ and the electrode surface, thereby synergistically improving current intensity and reaction efficiency.

To address the issue that the Zero Gap configuration electrolytic cell has limited internal space and cannot directly implement aeration for bromine (Br_2_) stripping, this study further designed a liquid flow system coupling internal reaction solution circulation with external aeration. The circulation rate of the internal reaction solution was controlled at 4–10 L/min, and an external aeration device was equipped (Figure 2b). On one hand, this coupled system can efficiently remove the generated Br_2_ through aeration, avoiding the inhibitory effect of Br_2_ accumulation in the electrolyte on the reaction. On the other hand, the constructed circulating flow field can scour the inner wall of the serpentine water channel, preventing salt crystallization (which may occur during the reaction) from blocking the flow channel. Additionally, it enhances the convective mass transfer of the electrolyte, further improving the electron transfer efficiency at the electrode interface.

Furthermore, to resist corrosion from bromine and acidic electrolyte and extend the service life of the electrolytic cell, an impact-resistant reinforced perfluorosulfonic acid cation exchange membrane was selected. This membrane ensures proton conduction efficiency while improving mechanical stability. The cell body was constructed using polytetrafluoroethylene (PTFE), and perfluororubber was used as the sealing gasket to resist the oxidative corrosion of bromine, ensuring long-term stable operation of the system.

### 3.3. Study on the Feasibility of Selective Oxidation of Bromide Ions

**Figure 3:**
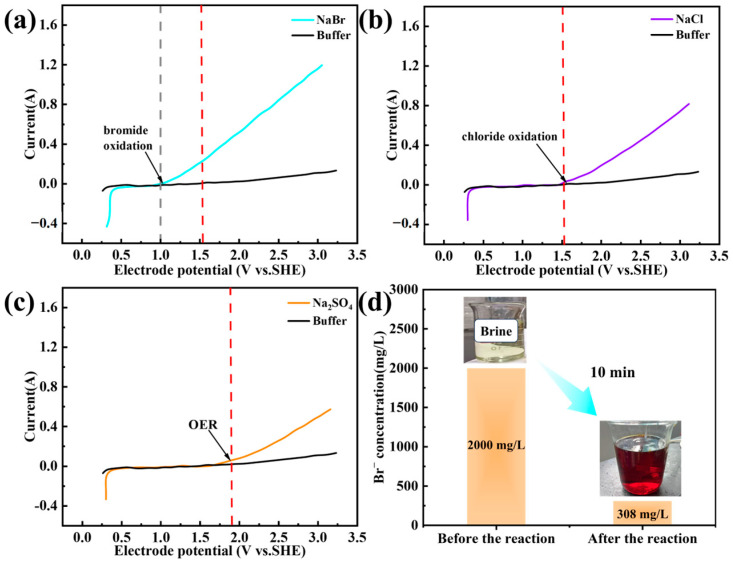
The results of Linear Sweep Voltammetry (LSV) measurements for (**a**) NaBr, (**b**) NaCl and (**c**) Na_2_SO_4_, (**d**) Br^−^ Conversion rated of simulated oil and gas field water (High Cl^−^, Low Br^−^) in a zero-gap electrolytic cell at 1.52 V (vs. Ag/AgCl).

By comparing the differences in the oxidation behavior of bromide ions (Br^−^) at different potentials, this study clarified the electrooxidation characteristics and kinetic laws of Br^−^, and systematically verified the electrooxidation kinetics of Br^−^ using the carbon cloth electrode and Zero Gap configuration electrolytic cell. The results of Linear Sweep Voltammetry (LSV) measurements are presented in Figure 3a, revealing that the oxidation processes of different ions exhibit distinct potential-dependent characteristics. For the NaBr solution, when the anode potential was lower than 1.0 V (vs. SHE), the voltammogram remained at a near-zero current level, indicating that the electrooxidation reaction of Br^−^ had not yet initiated. When the potential increased to 1 V (vs. SHE), a distinct inflection point appeared on the curve, and the cell current began to rise significantly. Meanwhile, the solution gradually turned pale yellow (a characteristic color of Br_2_), and bubbles were generated on the surface of the platinum sheet electrode, confirming the onset of the Br^−^ oxidation reaction (2Br^−^ → Br_2_ + 2e^−^). Furthermore, the standard redox potential of Br^−^ is 1.087 V (vs. SHE), resulting in an oxidation overpotential (η_Br_^−^) of −0.117 V. As the potential further increased to 1.52 V (vs. SHE), the current showed a linear upward trend, peaking at 0.2 A, while the solution color gradually deepened to dark red. This phenomenon indicates that the oxidation reaction of Br^−^ proceeded rapidly.

Meanwhile, for the comparative NaCl solution (Figure 3b), no significant current change was observed in the LSV curve when the anode potential was lower than 1.52 V (vs. SHE), and the characteristic yellow-green color of Cl_2_ (unique to chlorine gas) did not appear in the solution. When the potential exceeded approximately 1.52 V (vs. SHE), a current inflection point emerged on the curve, marking the initiation of the Cl^−^ oxidation reaction. Calculations based on the standard redox potential of Cl^−^ (1.358 V vs. SHE) show that its oxidation overpotential (η_Cl_^−^) reaches 0.162 V, which is significantly higher than that of Br^−^. This difference results in an actual oxidation potential difference of 0.55 V between Br^−^ and Cl^−^, which is much larger than the theoretical standard potential difference (0.271 V). This indicates that the carbon cloth electrode and Zero Gap configuration electrolytic cell can provide sufficient kinetic space for the realization of the selective oxidation of Br^−^.

Meanwhile, the results of our interference evaluation on the Oxygen Evolution Reaction (OER) showed that a Na_2_SO_4_ solution, which is difficult to electrooxidize, was selected as the working solution. Experiments revealed that when the anode potential was lower than 1.8 V (vs. SHE), only a weak current was generated (Figure 3c), and very few bubbles were produced on the electrode surface—indicating that the oxidation reaction of OH^−^ was significantly inhibited [30,31]. This is mainly attributed to the extremely low concentration of OH^−^ under acidic conditions ([OH^−^] = 10^−11^ mol/L) and the extremely high overpotential of OER (η > 1.0 V at 10 mA/cm^2^). Thus, within the potential range of 1.0–1.52 V (vs. SHE), the interference of water electrolysis side reactions on the Br^−^ oxidation process is negligible [32,33].

The above results collectively confirm that the range of 1.0–1.52 V (vs. SHE) is an effective potential window for the selective oxidation of Br^−^. Within this range, Br^−^ can be oxidized prior to Cl^−^ and OH^−^.

Based on the analysis results of the theoretical potential window, this study further screened the optimal operating potential that balances reaction efficiency and selectivity. The selection of the operating potential needs to meet three constraints. First, it needs to be higher than the critical activation potential of Br^−^ oxidation (0.97 V vs. SHE) to ensure sufficient reaction rate. According to the Butler-Volmer equation, a moderate increase in potential can exponentially enhance the reaction current density [34]. Second, it needs to be significantly lower than the initial oxidation potential of Cl^−^ (1.52 V vs. SHE) to reserve a safe margin, preventing Cl^−^ oxidation side reactions caused by electrode polarization fluctuations or changes in solution composition. Third, it needs to be lower than the potential at which the oxygen evolution reaction (OER) occurs significantly (1.8 V vs. SHE) to reduce the loss of current efficiency caused by water decomposition.

Considering comprehensively the kinetic advantages and selectivity risks, 1.52 V (vs. SHE) was finally selected as the operating potential. This potential is 0.55 V higher than the initial oxidation potential of Br^−^, which not only ensures sufficient reaction driving force but also maintains a safe distance from the initial oxidation potential of Cl^−^ and stays far from the region where OER occurs significantly.

To verify the applicability of the selected operating potential in actual brine environments (t), this study conducted constant-potential electrolysis experiments. For the experiments, 1 L of simulated oil and gas field water (Cl^−^ concentration is 3.4 mol/L, Br^−^ concentration is 60 mmol/L), with compositions based on global brine data in Table 1, was used as the anode working solution, and 2 L of acidic water (HCl, pH = 3) was used as the cathode working solution. A Zero Gap electrolytic cell was employed. The anode compartment was sealed to collect Br_2_, while the cathode compartment was open to the atmosphere for H_2_ discharge. Both compartments were circulated at a flow rate of 4 L/min to maintain solution homogeneity.

As shown in Figure 3d, after 10 min of continuous electrolysis, the cell current gradually decreased to its initial level with the consumption of Br^−^, and the solution color deepened to dark red. Ion chromatography (IC) detection results revealed that the Br^−^ conversion rate reached 92.3%, while the Cl^−^ concentration remained unchanged. This result directly confirms that at an operating potential of 1.52 V (vs. Ag/AgCl), the carbon cloth electrode can still achieve highly selective and efficient oxidation of Br^−^ in actual brine environments where the Cl^−^ concentration is much higher than that of Br^−^, effectively inhibiting the competitive oxidation reaction of Cl^−^.

### 3.4. Influence of Coexisting Cl^−^ on Electrooxidative Bromine Extraction

**Figure 4:**
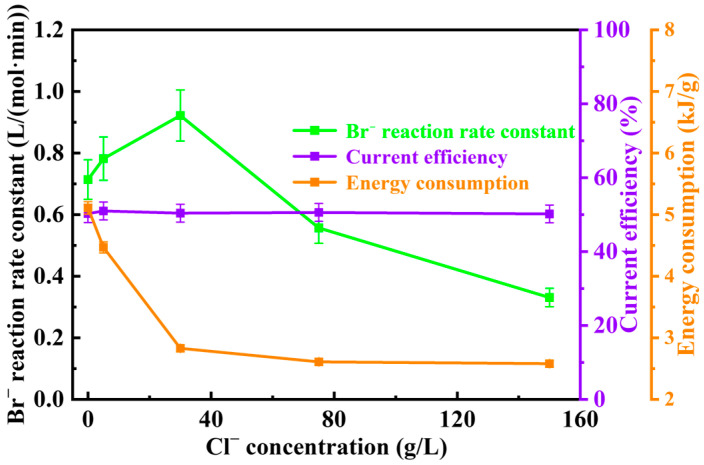
Effect of Cl^−^ concentration on the reaction rate constant of Br^−^, current efficiency, and specific energy consumption.

Since selective electrooxidative bromine extraction aims to achieve selective oxidation of Br^−^ in chloride-rich solutions by controlling the anode potential, the influence of coexisting Cl^−^ on this technology warrants investigation. Although the anode potential is precisely controlled, preventing Cl^−^ oxidation, Cl^−^ may still affect the process in other ways. Using an average Br^−^ concentration of 5 g/L as the experimental concentration and based on common Cl^−^ concentrations in brine, five concentration gradients of Cl^−^ were selected for the study: 0.0 g/L (no Cl^−^ added), 5 g/L (equal to Br^−^ concentration), 30.0 g/L (low concentration), 75.0 g/L (average concentration), and 150.0 g/L (high concentration). The results are shown in Figure 4.

Analysis of the results shows that at a Br^−^ concentration of 5 g/L, the reaction rate constant of Br^−^ initially increased and then decreased with increasing Cl^−^ concentration. When no Cl^−^ was added to the solution (0.0 g/L), the initial reaction rate constant was 0.714 L/(mol·min). When a small amount of Cl^−^ was added (5 g/L), the reaction rate constant rapidly increased to 0.782 L/(mol·min). As the Cl^−^ concentration further increased, the growth rate slowed. When the Cl^−^ concentration reached 30.0 g/L, the reaction rate constant increased to 0.922 L/(mol·min). With continued increase in Cl^−^ concentration, the reaction rate constant began to decrease rapidly. When Cl^−^ concentration reached the average level in underground brine (75.0 g/L), the rate constant dropped to 0.557 L/(mol·min). At the high Cl^−^ concentration level found in underground brine (150.0 g/L), the rate constant decreased to 0.331 L/(mol·min). The initial increase in the reaction rate constant may be related to the enhancement of the solution’s total electrical conductivity. The initial solution contained only 5 g/L Br^−^, resulting in relatively low total conductivity. Adding a small amount of Cl^−^ significantly increased the total conductivity of the solution, promoting the electrooxidation reaction and rapidly increasing the rate constant. As Cl^−^ in the solution continued to increase, the promoting effect due to increased conductivity theoretically persisted. However, the subsequent slowdown and eventual decrease in the rate constant may be related to other effects of Cl^−^. Since Cl^−^ is not oxidized at this potential, its inhibitory effect on the electrooxidation reaction may be associated with adsorption competition between Cl^−^ and Br^−^ on the titanium mesh electrode surface [33].

Although Cl^−^ inhibits Br^−^ electrooxidation due to adsorption competition on the electrode surface, the specific energy consumption continuously decreased as Cl^−^ was added and the total conductivity of the solution increased. Without Cl^−^ added (0.0 g/L), the specific energy consumption was highest at 5.11 kJ/g. When Cl^−^ concentration increased to 150.0 g/L, it reached the lowest value of 2.58 kJ/g. Without Cl^−^ added (0.0 g/L), the total conductivity of the solution was very low, resulting in a higher cell voltage during the reaction. Additionally, since the reaction rate was relatively low at this point, Br_2_ production was also low, leading to high energy consumption per unit mass of Br_2_ product. As Cl^−^ was continuously added to the solution, the total conductivity increased, and the cell voltage decreased accordingly. From the trend of the reaction rate constant with Cl^−^ concentration, it is known that before Cl^−^ concentration reached 30.0 g/L, the reaction rate also increased with increasing Cl^−^ concentration, leading to higher Br_2_ production. Consequently, the specific energy consumption decreased rapidly. When Cl^−^ concentration continued to increase beyond 30.0 g/L, the decrease in cell voltage became smaller, and the reaction rate began to decrease with increasing Cl^−^ concentration, leading to reduced Br_2_ production. This caused the decline in specific energy consumption to lessen. Current efficiency remained relatively stable regardless of Cl^−^ concentration, indicating minimal influence from chloride levels.

In summary, coexisting Cl^−^ exhibits both promoting and inhibitory effects on Br^−^ electrooxidation. The promotion originates from the increase in the solution’s total electrical conductivity with rising Cl^−^ concentration, while the inhibition stems from adsorption competition between Cl^−^ and Br^−^ on the titanium mesh electrode surface. In the initial stage of increasing Cl^−^ concentration, the promoting effect dominates, and the reaction rate increases rapidly with Cl^−^ addition. After the Cl^−^ concentration exceeds the common level in underground brine (>30.0 g/L), the inhibitory effect begins to outweigh the promoting effect, and the reaction rate decreases rapidly with further increases in Cl^−^ concentration. The decrease in specific energy consumption with increasing Cl^−^ concentration partially compensates for the adverse effects such as decreased reaction rate and increased time cost caused by high Cl^−^ levels. The current efficiency is relatively unaffected by Cl^−^ concentration.

### 3.5. Influence of Coexisting Carbonate Ions on Electrooxidative Bromine Extraction

After investigating the influence of coexisting Cl^−^, another significant coexisting anion worthy of study is CO_3_^2−^. Although CO_3_^2−^ is difficult to oxidize within the potential range discussed in this study and does not participate in competitive oxidation with Br^−^ like Cl^−^, its content in concentrated brine and common underground brine types is second only to Cl^−^ among all common anions. Therefore, the presence of CO_3_^2−^ significantly impacts solution properties such as electrical conductivity and total dissolved solids, thereby influencing physical migration processes and electrochemical reactions occurring in the solution [35]. Furthermore, as an anion, CO_3_^2−^ migrates towards the electrode surface alongside Br^−^ during electrification. How CO_3_^2−^ affects the structural properties on the solution side of the electrode-solution interface and consequently impacts Br^−^ electrooxidation is also worth exploring. Therefore, the influence of the presence and concentration of CO_3_^2−^ on Br^−^ electrooxidation became another key research focus following the study on Cl^−^.

**Figure 5:**
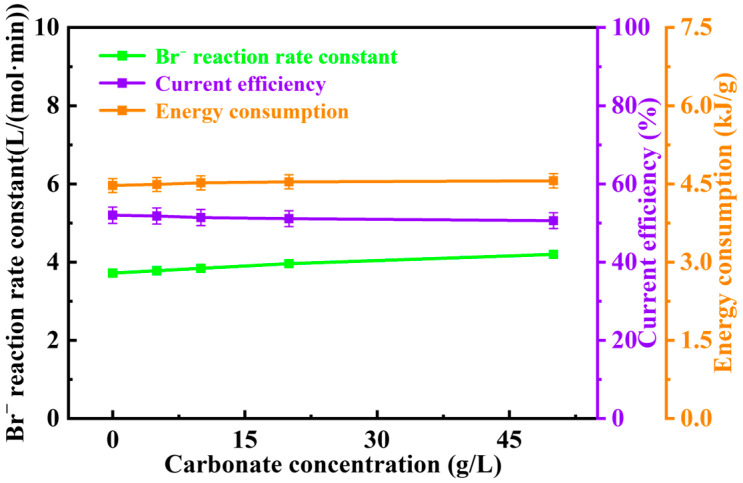
Effect of CO_3_^2−^ concentration on the reaction rate constant of Br^−^, current efficiency, and specific energy consumption.

Since this study focuses on Br^−^ electrooxidation technology in chloride-rich solutions, i.e., selective oxidation of Br^−^, investigating the influence of CO_3_^2−^ alone in the absence of Cl^−^ is meaningless. Consequently, the study on the effect of CO_3_^2−^ presence and concentration on Br^−^ electrooxidation was conducted based on a Cl^−^ concentration of 30 g/L. Referring to common CO_3_^2−^ concentrations in concentrated and underground brines, concentrations of 0.00 g/L, 5 g/L (low concentration), 10.00 g/L (medium concentration), 20.00 g/L (high concentration), and 50.00 g/L (very high concentration) were selected for investigating the effect of CO_3_^2−^ concentration. Additionally, similar to the Cl^−^ influence study, calcium and magnesium salts were avoided in solution preparation, replaced by sodium salts, to preclude potential influence from solution hardness. Reaction rate constant, current efficiency, and specific energy consumption were again selected as reference indicators to examine the influence of CO_3_^2−^ presence and concentration on Br^−^ electrooxidation. The changes in reaction rate constant, current efficiency, and specific energy consumption with increasing CO_3_^2−^ concentration from zero are shown in Figure 5.

The results show that the reaction rate constant of Br^−^ electrooxidation increased linearly and slowly with increasing CO_3_^2−^ concentration, exhibiting the same trend as observed when Cl^−^ concentration increased beyond 30 g/L. However, its growth rate was much lower than that observed with increasing Cl^−^ concentration. Without CO_3_^2−^ added (0.00 g/L), the reaction rate constant was minimum at 1.232 L/(mol·min). It reached a maximum of 1.348 L/(mol·min) at an CO_3_^2−^ concentration of 20.00 g/L. The promoting effect of CO_3_^2−^ on Br^−^ electrooxidation may also be attributed to the increase in the solution’s total electrical conductivity caused by adding CO_3_^2−^. To verify this hypothesis, a validation experiment was designed: increasing the CO_3_^2−^ concentration in the solution to 50.00 g/L and investigating the change in reaction rate constant. If the reaction rate constant further increased, it would support the hypothesis that increased total conductivity promotes Br^−^ electrooxidation. The result shows that as CO_3_^2−^ concentration increased to 50.00 g/L, the reaction rate constant of Br^−^ electrooxidation continued to increase linearly with CO_3_^2−^ concentration, thus validating the hypothesis. The much lower rate of increase in the Br^−^ reaction rate constant with CO_3_^2−^ concentration compared to that with Cl^−^ concentration also indicates that the promoting effect of CO_3_^2−^ on Br^−^ electrooxidation is far weaker than that of Cl^−^. This phenomenon occurs because CO_3_^2−^ has a larger molecular weight than Cl^−^. When adding the same mass of the two ions to the solution, the number of dissociated CO_3_^2−^ ions is much less than that of Cl^−^ ions. Consequently, CO_3_^2−^ contributes less to enhancing the solution’s total conductivity and exerts a weaker promoting effect on Br^−^ electrooxidation at the same mass concentration.

The specific energy consumption for Br^−^ electrooxidation also increased linearly and slowly with increasing CO_3_^2−^ concentration, following the same trend as observed when Cl^−^ concentration increased beyond 30 g/L. Without CO_3_^2−^ added (0.00 g/L), the specific energy consumption was lowest at 4.28 kJ/g. It reached a maximum of 4.37 kJ/g at an CO_3_^2−^ concentration of 20.00 g/L. Unlike the study on Cl^−^‘s influence, the baseline solution in this study contained 30 g/L Cl^−^. Even without added CO_3_^2−^, sufficient ions were present to provide the current required for the Br^−^ electrooxidation reaction at the electrode surface. Therefore, issues like high cell voltage and low reaction rate did not occur, nor did high energy consumption.

The current efficiency for Br^−^ electrooxidation decreased linearly and slowly with increasing CO_3_^2−^ concentration, consistent with the trend observed when Cl^−^ concentration increased beyond 30 g/L. Without CO_3_^2−^ added (0.00 g/L), the current efficiency was highest at 53.1%. It decreased to a minimum of 52.3% at an CO_3_^2−^ concentration of 20.00 g/L. Similar to the decrease in current efficiency with increasing Cl^−^ concentration, the reason for the decrease with increasing CO_3_^2−^ concentration may also be enhanced water electrolysis. The increase in total solution conductivity, while promoting Br^−^ electrooxidation, also promotes water electrolysis. This leads to a larger proportion of the reaction current being consumed by water electrolysis, reducing the current efficiency for the target reaction. The linear increase in specific energy consumption may also be caused by enhanced water electrolysis, as water electrolysis consumes more energy, leading to increased energy consumption per unit mass of the Br_2_ product.

Through the investigation of the influence of coexisting anions on Br^−^ electrooxidation, it was found that Cl^−^ not only fails to inhibit Br^−^ electrooxidation but also exhibits a strong promoting effect due to its enhancement of the solution’s total conductivity. CO_3_^2−^ also shows a promoting effect on Br^−^ electrooxidation due to its contribution to total conductivity. However, due to its relatively larger molecular weight, it dissociates fewer ions at the same mass concentration, resulting in a weaker promoting effect. Furthermore, when the total ion concentration is sufficiently high, the enhancement of water electrolysis adversely affects the current efficiency of the target reaction and the specific energy consumption. The increased extent of water electrolysis reduces the proportion of the target reaction in the total reaction current and increases the energy consumed by water electrolysis per unit product, thereby raising the cost of the electrooxidation method.

### 3.6. Influence of Hardness Ions (Ca^2+^ and Mg^2+^) on Electrooxidative Bromine Extraction

Following the investigation of coexisting anions, the influence of another major category of ions present in concentrated or underground brine, hardness ions (Ca^2+^, Mg^2+^), is also worth exploring. High hardness is a significant characteristic of concentrated and underground brine, originating from the enriched calcium and magnesium ions [36]. The impact of this high hardness caused by these ions on the electrooxidation of Br^−^ at the electrode surface remains to be studied. Additionally, although calcium and magnesium ions do not participate in Br^−^ electrooxidation, their effect on the structural properties on the solution side of the electrode-solution interface and consequently on the Br^−^ electrooxidation reaction is also worthy of discussion.

**Figure 6:**
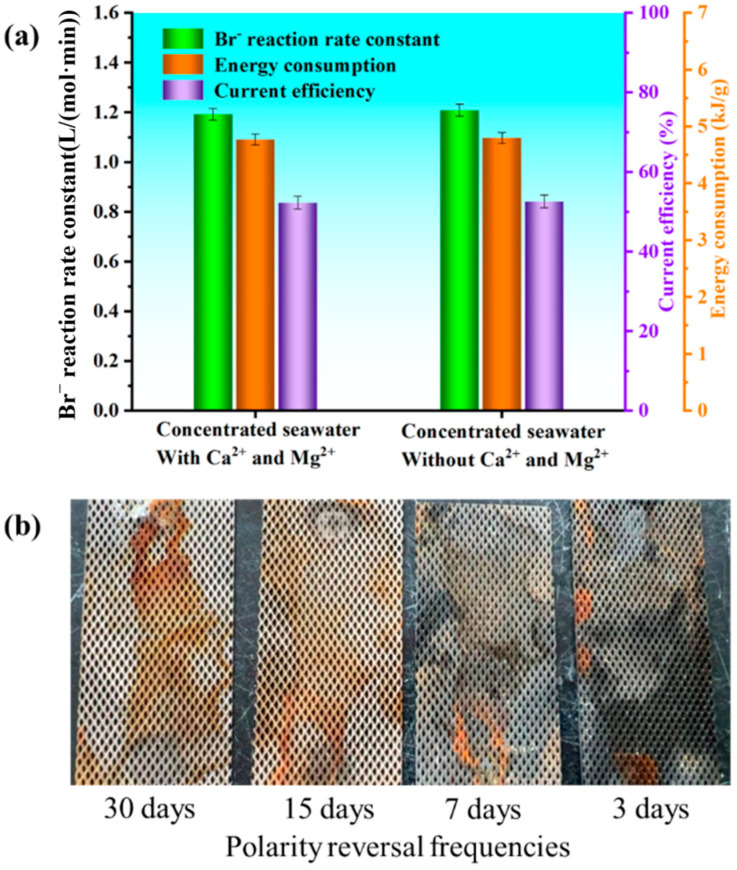
(**a**) Comparison of Br^−^ reaction rate constant, current efficiency, and specific energy consumption in concentrated brine with and without the presence of calcium and magnesium ions, (**b**) morphology of electrode scaling in high-hardness water under different polarity reversal frequencies.

Therefore, this study employed a direct comparison metho. Concentrated brine containing calcium and magnesium ions and concentrated brine without these ions were subjected to electrooxidation treatment separately. By comparing various reference indicators of the oxidation reaction, the influence of hardness ions on the technology was determined. Reaction rate constant, current efficiency, and specific energy consumption were still selected as reference indicators. In the electrooxidation experiments, a real concentrated brine containing calcium and magnesium ions was investigated. For comparison, a synthetic brine with the same ionic composition (5 g/L CO_3_^2−^, 30 g/L Cl^−^, and 5 g/L Br^−^) but without calcium and magnesium ions was also employed. A comparison of the three reference indicators between the two is shown in Figure 6a. Under conditions with or without the presence of calcium and magnesium ions, the reaction rate constant, current efficiency, and specific energy consumption for Br^−^ electrooxidation in concentrated brine were very similar. This indicates that the presence of hardness ions has minimal impact on the selective electrooxidation of Br^−^. With calcium and magnesium ions present, the reaction rate constant was 1.181 L/(mol·min), current efficiency was 51.0%, and specific energy consumption was 4.61 kJ/g. Without calcium and magnesium ions, the values were 1.242 L/(mol·min), 51.2%, and 4.64 kJ/g, respectively. The negligible difference in the reference indicators with or without calcium and magnesium ions demonstrates that the selective electrooxidation technology is minimally affected by the hardness of concentrated or underground brine, highlighting its excellent applicability.

However, Then, high concentrations of Ca^2+^ and Mg^2+^ ions may lead to scaling on the electrode surface. Figure 6b presents the scaling characteristics of electrodes in high-hardness wastewater under varying polarity reversal (polarity inversion) frequencies. The electrode substrate is a metal mesh with a diamond-pore structure. When the polarity reversal frequency is low, the electrode surface is covered by a substantial, yellowish-brown, and dense scaling layer, which exhibits extensive distribution and considerable thickness, leading to high coverage on the electrode surface. As the polarity reversal frequency increases (sequentially, once every 2 weeks, once per week), the scaling layer on the electrode surface gradually fades in color, reduces in coverage area, and thins out. When the polarity reversal frequency is raised to once every 3 days (the rightmost electrode), only a small amount of dispersed scale exists on the electrode surface, and the electrode remains relatively clean overall.

Electrode scaling stems from the electrochemically induced crystallization and deposition of scaling cations (Ca^2+^, Mg^2+^) in high-hardness wastewater on the electrode surface, and polarity reversal regulates scaling layer formation and detachment by altering the electric field distribution and ion migration direction on the electrode surface: at low polarity reversal frequencies, the electrode maintains a single polarity for a long time, allowing scaling ions to migrate and deposit continuously on its surface, and the lack of timely reverse peeling force leads to the gradual thickening and densification of the scaling layer; while at higher frequencies, the polarity-switching cycle shortens, the charge polarity of the electrode surface flips during each reversal, which not only inhibits the continuous directional deposition of scaling ions but also exerts an electrochemical peeling effect on preformed scaling layers, thus effectively reducing scaling accumulation and significantly decreasing the electrode scaling degree with the increase in polarity reversal frequency.

### 3.7. Influence of Organic Matter Concentration on Electrooxidative Bromine Extraction

Dissolved organic matter (DOM) is commonly present in practical concentrated brines. Such substances may not only consume active oxidizing species during electrolysis but also occupy active sites through adsorption or polymerization on the electrode surface, thereby affecting electrode catalytic activity and the efficiency of selective Br^−^ oxidation [37,38]. Currently, research on the influence mechanism of organic matter concentration on the electrooxidative bromine extraction process from concentrated brine remains relatively limited. Systematic investigation of its impact is of significant theoretical and practical value for optimizing bromine extraction parameters and enhancing the treatment efficiency of real brines.

**Figure 7:**
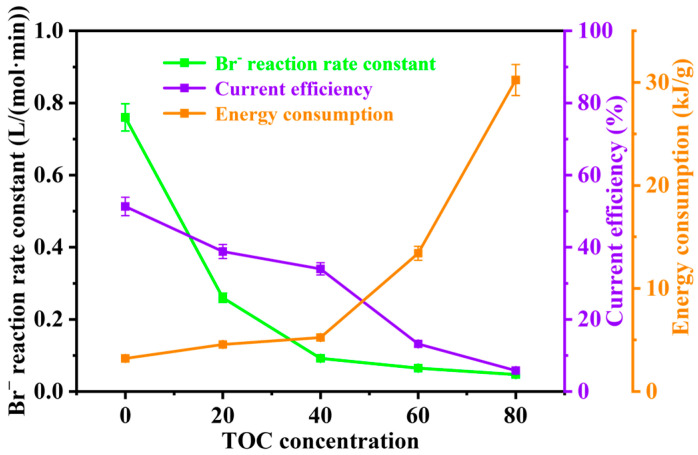
Effect of Organic Matter Concentration on Br^−^ Reaction Rate Constant, Current Efficiency, and Specific Energy Consumption.

These results demonstrate that organic matter concentration in brine significantly impacts the electrooxidative bromine extraction reaction (Figure 7), primarily through four mechanistic pathways: competitive adsorption for active sites on the electrode surface, where ethylene glycol as a polar organic compound preferentially occupies active sites on the carbon cloth electrode, hindering effective Br^−^ adsorption and electron transfer; electrochemical oxidation competition due to ethylene glycol’s lower oxidation potential compared to the Br^−^ oxidation onset potential (1.0 V vs. SHE), enabling its preferential anodic oxidation and reducing current efficiency for Br^−^ oxidation; alterations in solution physicochemical properties caused by interactions between ethylene glycol, water molecules, and inorganic ions that diminish ionic strength and conductivity, coupled with potential chemical reactions between ethylene glycol oxidation products and Br_2_ that reduce detectable Br^−^ concentration; and mass transfer suppression wherein ethylene glycol elevates solution viscosity, decreasing Br^−^ mass transfer diffusion rates toward the electrode surface, ultimately causing significant reaction deceleration due to insufficient reactant supply when mass transfer rates fail to match electrode catalytic kinetics.

## 4. Conclusions

This study confirms the feasibility and high efficiency of electrochemical chlorine-free bromine extraction technology, addressing the development needs of high-concentration bromine brine from underground oilfields. Leveraging the standard redox potential difference between Br^−^ and Cl^−^ (0.271 V), the effective potential window for selective Br^−^ oxidation was determined as 1.0–1.52 V (vs. SHE) via linear sweep voltammetry. Within this window, Br^−^ can be oxidized preferentially over Cl^−^ and OH^−^. Experiments simulating high-chloride, low-bromide brine demonstrated a Br^−^ conversion rate of 92.3% with no Cl_2_ generation at this potential. Kinetic studies revealed that the reaction follows first-order kinetics, and current intensity exhibits a significant positive correlation with Br^−^ concentration, providing a quantitative basis for monitoring the reaction progress.

Process optimization significantly enhanced bromine extraction performance. The self-designed zero-gap electrolyzer with carbon cloth as the anode reduced the reaction time by over 75% compared to a traditional H-type cell, oxidizing over 90% of Br^−^ within 12 min. Investigation of coexisting ions revealed that low concentrations of Cl^−^ (≤30 g/L) promote the reaction by enhancing conductivity, while high concentrations inhibit it due to adsorption competition. CO_3_^2−^ exerts a weak promoting effect, and Ca^2+^/Mg^2+^ have negligible impact on the reaction, demonstrating the technology’s good applicability to complex brines. Organic matter (exemplified by ethylene glycol) induces near-complete reaction cessation and a drastic decline in bromine recovery efficiency at concentrations exceeding 80 mg/L, primarily through competitive electrode adsorption, preferential oxidation reactions, and increased solution viscosity. This work systematically elucidates the effects of diverse ions and organic compounds on electrooxidative bromine extraction, establishing critical foundations for optimizing process parameters and designing robust equipment. The technology effectively resolves inherent safety hazards and energy consumption issues of traditional chlorine gas methods, provides technical support for sustainable exploitation of high-concentration bromine resources, and serves as a methodological reference for selective extraction of target ions from complex brine systems.

## Figures and Tables

**Table 1:** Main components and their concentrations of major global oil and gas field water.

Oil and Gas Field Water	Na^+^ (mg/L)	K^+^ (mg/L)	Ca^2+^ (mg/L)	Mg^2+^ (mg/L)	Cl^−^ (mg/L)	Br^−^ (mg/L)
Gas field water in the Sichuan Basin	92,200	52,000	4280	2330	19,807	2590
Oil field water in the Qaidam Basin	11,140	4542	12,888	1909	13,902	107
Oil field water in the Jianghan Basin	103,660	9220	16,400	30	198,800	202
Oil field water in Mississippi State, USA	55,200	7560	45,200	4240	18,550	2100
Oil field water in Arkansas State, USA	64,200	11,100	44,600	3250	20,270	5440
Oil field water in Texas State, USA	69,300	6390	28,300	2730	17,070	2370
Oil field water in the Udachinaya Oilfield, Russia	12,098	15,656	61,202	10,983	17,909	4910
Oil field water in the Balagankinskaya Oilfield, Russia	2775	15,141	11,988	9024	20,587	5135
Oil field water in the Kuturminsk Oilfield, Russia	8764	19,843	96,021	9303	16,169	4252

## Data Availability

The original contributions presented in this study are included in the article/Supplementary Material. Further inquiries can be directed to the corresponding author.

## Supplementary Materials

The following supporting information can be downloaded at: https://www.mdpi.com/article/10.3390/ma19050850/s1, Figure S1: Schematic diagram of the experimental setup for LSV.
