# Supplementary material for: Synergistic Enhancement of Electrochemical-Oxidative Chlorine-Free Bromine Extraction from Oil and Gas Field Water by Zero-Gap Electrolyzer and Carbon Cloth Electrode: A Study on Efficient, Selective Extraction and Resistance to Other Ions
Source: Materials (Basel). 2026 Feb 25;19(5):850. doi: 10.3390/ma19050850 (PMC12985701; doi:10.3390/ma19050850)
Supplement: Supplementary file 1 [file materials-19-00850-s001.zip › materials-3946622-supplementary.pdf]

Supplementary Materials

Synergistic Enhancement of Electrochemical-Oxidative Chlorine-Free Bromine Extraction from Oil and Gas Field Water by Zero-Gap Electrolyzer and Carbon Cloth Electrode: A Study on Efficient, Selective Extraction and Resistance to Other Ions

Shiyong Zhou[a], Rong Ji[b], Yuan Li[a*]

[a] Hubei Key Laboratory of Mineral Resources Processing and Environment, Key Laboratory of Green Utilization of Critical Non-metallic Mineral Resources, Ministry of Education, Wuhan University of Technology, 122 Luoshi Road, Wuhan 430070, China

[b] School of Metallurgy and Environment, Central South University, Changsha 410083, China

This supporting information contains:
Number of Pages:1
Number of Figures:1

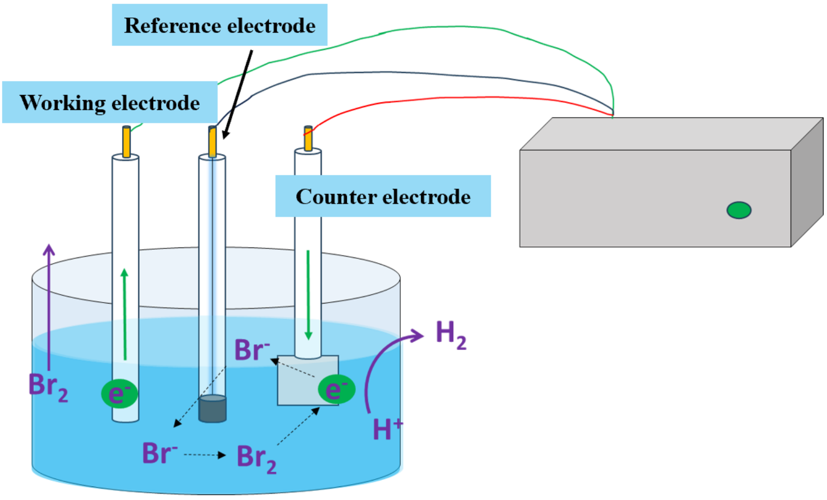

Fig. S1. Schematic diagram of the experimental setup for LSV.
